# Lumbar punctures based on computerized tomography scans combined with precise calculations: a new lumbar puncture technique for spinal muscular atrophy patients with scoliosis

**DOI:** 10.3389/fneur.2025.1654310

**Published:** 2025-08-13

**Authors:** Hongyuan Li, Juan Du, Xi Chen, Hanbing Lu, Yan Jiao, Shuyu Dong

**Affiliations:** ^1^Department of Neurology, Xuzhou Central Hospital No. 199, Xuzhou, Jiangsu, China; ^2^Department of Obstetrics and Gynecology, Xuzhou Central Hospital No. 199, Xuzhou, Jiangsu, China

**Keywords:** lumbar puncture, CT scan, precise calculation, scoliosis, spinal muscular atrophy

## Abstract

**Objective:**

We report our experience performing lumbar punctures using a novel technique in (Spinal muscular atrophy) SMA patients with scoliosis.

**Methods:**

A retrospective case series was conducted at the Department of Neurology at Xuzhou Central Hospital from January 2022 to August 2024. Patients diagnosed as SMA combined with scoliosis underwent 58 lumbar punctures using a novel guidance technique. Data on first pass success rate, needling time, using of sedation and adverse events (AEs) were collected and analyzed.

**Results:**

A total of 58 successful lumbar punctures were performed on six SMA patients with scoliosis using this novel technique. Most patients achieved first pass success for each LP, except for one patient. The media first LP needling time was 70.5 s (range: 45 s−150 s). For subsequent procedures, the needling time was shorter than for the first LP in all patients. Apart from one instance of back pain and one case of post-puncture headache, no other AEs were observed.

**Conclusion:**

Lumbar punctures guided by a single CT scan combined with precise calculations can be broadly and safely applied for intrathecal injection in patients with scoliosis.

## Introduction

Spinal muscular atrophy (SMA) is an autosomal recessive disorder marked by progressive symmetrical muscle weakness and atrophy, most often affecting the proximal limbs and trunk (1). SMA results from the homozygous deletion of exon 7 or mutation in the *SMN1* gene (2). This leads to reduced expression or loss of function of the SMN protein, causing degeneration of motor neurons in the anterior horn of the spinal cord and medulla oblongata. It also causes morphological and functional changes in neuronal synapses and neuromuscular junctions, leading to typical neurogenic muscle atrophy (3). The incidence in European and American newborns is ~1 in 10,000, with a carrier frequency of 1 in 40 to 1 in 50 (4). Currently, the approved disease-modifying treatments for SMA in China include Nusinersen (5, 6) and Risdiplam (7). Nusinersen is a chemically modified, fixed-sequence antisense oligonucleotide that enhances the production of full-length SMN protein by modifying SMN2 pre-messenger RNA splicing (8). Due to its high molecular weight, Nusinersen cannot cross the blood–brain barrier, requiring administration into the cerebrospinal fluid through intrathecal injection (9).

However, scoliosis in SMA patients complicates traditional lumbar puncture (LP) procedures. Approximately two-thirds of SMA patients develop progressive scoliosis (10), with severe scoliosis being major complications (11).

The intrathecal administration of nusinersen poses unique challenges for patients with severe scoliosis (12). The main LP techniques for patients with scoliosis include computed tomography (CT)-guided, fluoroscopy-guided, and ultrasound-guided LP. Nusinersen is administered six times in the first year and three times annually thereafter (2). CT- and fluoroscopy-guided LPs are associated with complications from cumulative radiation exposure (13). Ultrasound-guided LP requires high technical skills and rich experience. Some patients have to be referred to the department of anesthesiology for intrathecal injection (14). Therefore, exploring a novel LP technique that allows neurologists to successfully perform intrathecal injections in SMA patients with scoliosis independently is necessary.

In this study, we propose a new approach combining a single CT scan with precise calculations for performing LP in SMA patients with scoliosis. Patient data are presented to demonstrate the reliability and safety of this approach.

## Materials and methods

### Patients

SMA patients with scoliosis who were admitted to the Department of Neurology at Xuzhou Central Hospital between January 2022 and August 2024 were included in this study. Patients with contraindication to LP were excluded from this study. Intrathecal Nusinersen injections were administered using a single CT scan combined with precise calculation-guided LP.

### Lumbar puncture procedures

#### Spine CT scan

**Figure 1:**
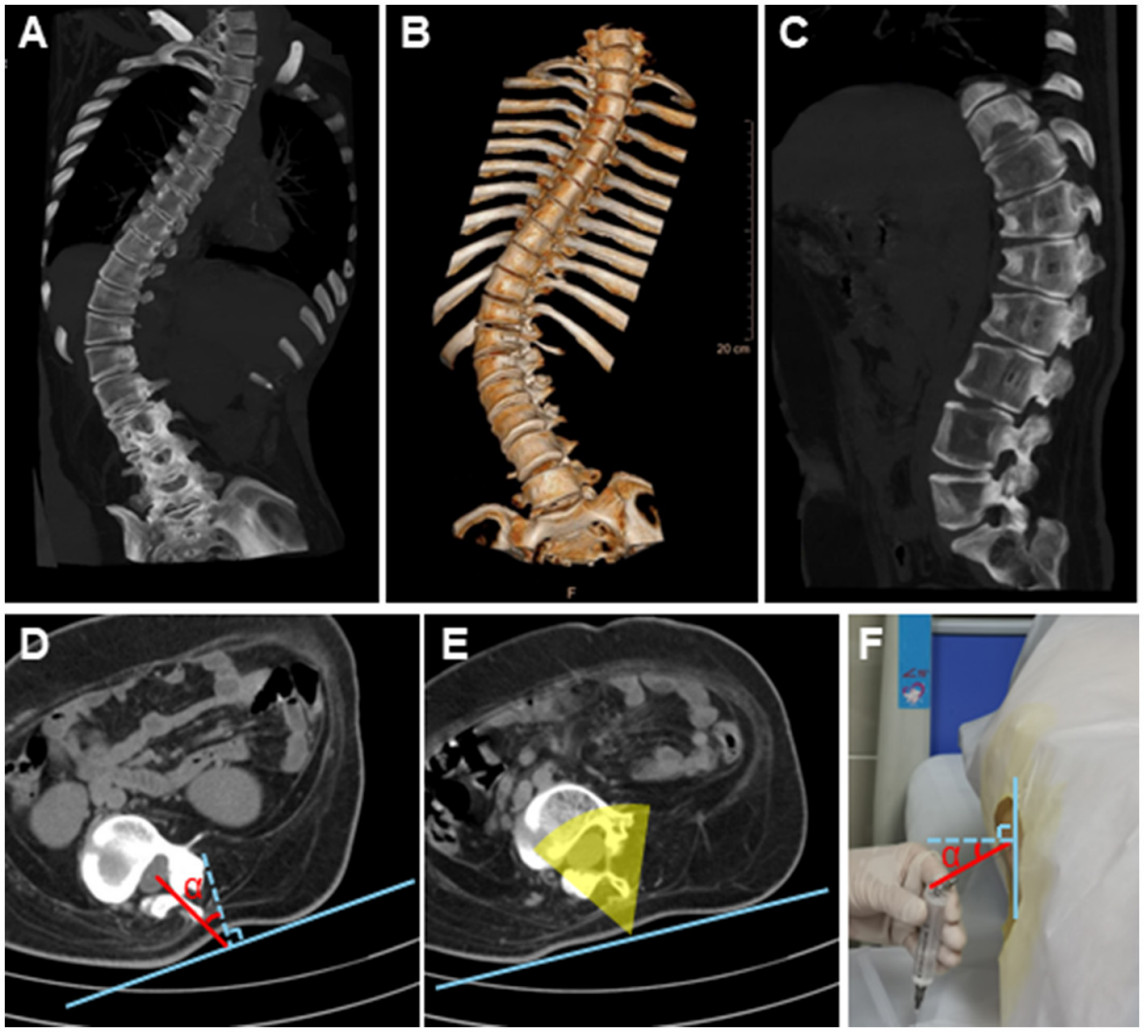
Plain CT scan combined with precise calculation guided lumbar puncture in an SMA patient with scoliosis **(A)** Anteroposterior spine radiograph shows a curve in the lumbar spine. **(B)** 3D reconstruction with volume rendering technique of the spine. **(C)** Lateral spine radiograph shows the intervertebral space of lumbar spine clearly. **(D)** Transverse dimension scan at the level of L3–L4. The blue line is the auxiliary line, the blue dotted line is the perpendicular line, the red line merely represents a simulated puncture path, not the practical one, and the red angle is angle α. **(E)** Transverse dimension scan at the level of L4–L5. The yellow sector represents the possible damage area under traditional lumbar puncture methods. **(F)** The patient is in left decubitus position.

Spine CT plain scan was operated (Figures 1A–E). CT scan of anteroposterior spine 3D volume rendering was used to measure cobb's angle (Figures 1A–C).

#### Precise calculation according to the cross-sectional images

The L3–L4 space (Figure 1D) was typically chosen as the optimal puncture path due to its largest intervertebral space. L4–L5 (Figure 1E) was the second option. Mapping and calculations were performed using PACS software. First, the puncture path was marked: the puncture point was where the spinous process extension intersected the skin, the target point was the center of the dural sac, and the line connecting these points formed the puncture path. Second, an auxiliary line was drawn at the puncture point, parallel to the coronal plane, followed by a perpendicular line. Third, the depth of the puncture path and its angle (called angle α) relative to the perpendicular line were measured (Figure 1D). It should be noted that the red line in Figure 1D did not represent the practical puncture path.

#### Intrathecal injection

Before the first lumbar puncture, we attached ECG electrode pads to the marked sites and then performed a CT scan. The metal protrusions on the electrode pads are easily identifiable on CT images, enabling rapid clarification of the exact level of the marked intervertebral space. Photographs of the skin puncture site were taken for documentation, and the distance from major body surface landmarks was measured. For subsequent punctures, there is no need for repeated CT scans. By referring to the photographs, the L3/4 intervertebral space can be quickly identified and located. We used the line connecting the bilateral posterior superior iliac spines to place the patients' back perpendicular to the bed surface. The puncture point was marked on the surface of the body (Figure 1F). It was needed to adjust the orientation of the needle according to the angle α and the angle toward the skull to avoid the needle tip meeting the lamina, and then lumbar puncture was performed as previously described (14). A 22G lumbar puncture needle was used.

### Variables collected as follows

*First pass success*, defined as the needle entering the dural sac successfully without another attempt.

*Number of attempts*, defined as the number of times the skin was punctured.

*Needling time*, defined as the amount of time measured in second from the needle contact with skin to CSF outflowing.

Whether *sedation* was recorded.

*Adverse events* were also collected.

## Results

### Patient characteristics

Six SMA patients with scoliosis were included in the study. The clinical characteristics of these patients were presented in Table 1. A total of 58 LPs were performed for intrathecal injections of Nusinersen. Among the patients, four were type 2 SMA and two were type 3 SMA. All patients were non-sitters now. The median age at diagnosis was 8 years (range: 3–11), and the median diagnostic delay was 5.5 years (range: 1–9). The median age at initial Nusinersen treatment was 29 years (range: 9–36), with a median treatment delay was 27.5 years (range: 8–36). All patients underwent a plain CT scan of the lumbar spine before their first LP. Patients 5 and 6 had moderate scoliosis, and patient 1–4 had severe scoliosis (15). Most patients have thoracolumbar curves, except for patient 2, which was classified as thoracolumbar/lumbar-main thoracic curve according to Lenke's classification criteria (16).

**Table 1:** The basic clinical data of SMA patients in this study.

**Pt**	**1**	**2**	**3**	**4**	**5**	**6**
Age of treatment initiation (years)	27	31	36	31	9	9
Gender	M	M	M	F	M	F
Age of onset (months)	18	18	10	12	14	8
Age of diagnose (years)	7	9	11	5	3	10
Diagnosis delay (years)	4.5	6.5	9	3	1	8
Treatment delay (years)	25	30	36	30	9	8
SMA type	3	3	2	2	2	2
Cobb's angle	60°	90°	50°	120°	40°	30°

M, male; F, female.

The characteristics and outcomes of lumbar punctures in SMA patients with scoliosis, based on precise calculations were presented in Table 2. Each patient underwent a midline LP at the L3–L4 level after comprehensive consideration. The media measured α angle was 15° (range: 13°-30°) and the measured depth was 4.3 cm (range: 3.5 cm−5.9 cm). The actual depth of each LP was shallower than the measured value. However, Cobb's angle did not appear to be associated with the rotation angle, defined as α angle. Most patients had first pass success during every LP, except for patient 4, who required three attempts at the first time. The media first LP needling time was 70.5 s (range: 45 s−150 s). After the first LP, needling times for each patient were shorter, as shown in Table 2. None of the patients required sedation.

**Table 2:** The characteristics and outcomes of lumbar punctures in SMA patients with scoliosis.

**Pt**	**Level of LP**	**Angle α**	**Measured depth**	**Lateral position**	**Actual puncture angle**	**Actual depth**	**First pass success/ Number of attempts**	**First LP needling time (s)**	**Needling time except first (s) Median (IQR)**	**sedation**	**AEs**
1	L3–L4	30°, right	4.5 cm	Left	30°	4.3 cm	11/11	125	38.5 (36.25,39.75)	N	N
2	L3–L4	13.5°, left	3.93 cm	Right	14°	3.8 cm	10/10	66	36 (35, 37)	N	N
3	L3–L4	15°, left	4.4 cm	Right	15°	4.1 cm	10/10	75	39 (36, 42)	N	N
4	L3–L4	15°, left	5.9 cm	Right	15°	5.2 cm	7/9	150	43 (37, 44.5)	N	Back pain
5	L3–L4	13°, right	4.2 cm	Left	13°	4.0 cm	8/8	45	35 (34.5, 39)	N	N
6	L3–L4	13°, right	3.5 cm	Left	13°	4.0 cm	10/10	51	31 (28, 32)	N	Post puncture headache

Angle α, the rotation angle of the LP targeted interspace of adjacent vertebral bodies; Measured depth, the depth from the presumed puncture point on the skin to the center of the sac; Actual depth, the depth of injection from the actual puncture point to the CSF outflow.

Only patient 4 and 6 had back pain and post puncture headache once relatively, which were relieved after several hours rest. No other adverse events (AEs) were reported.

## Discussion

Lumbar puncture is technically challenging in patients with scoliosis, a spinal deformity consisting of lateral curvature and vertebrae rotation (17). Traditional lumbar puncture carries a high risk of injury to abdominal organs, such as intestines and kidneys. Optimized lumbar puncture techniques have been developed specifically for patients with scoliosis. Currently, commonly used methods include ultrasound-guided (18–20), CT-guided (21, 22), and fluoroscopy-guided lumbar punctures (23, 24).

Despite significantly improving success rate in scoliosis, these lumbar puncture techniques still present disadvantages and challenges. Ultrasound-guided lumbar puncture is the most commonly used due to its advantage of causing minimal damage to the human body. However, color ultrasound provides poor image of bone tissue (25), requiring careful identification of the dural sac during the procedure. During operation, the color Doppler ultrasound probe occupies much of the vision field, reducing puncture flexibility. Not all neurologists are proficient in using ultrasound machines (26). X-ray provides superior bone imaging; however, fluoroscopy-guided lumbar puncture requires prolonged exposure, increasing the risks of radiation damage to both operators and patients. In addition, the operator must be skilled in using the fluoroscopy C-arm machine (14). SMA patients with scoliosis often have vertebral rotation in different spinal segments. In previous studies, spinal rotation is typically graded by angle alone, and the angle value is not used for lumbar puncture guidance (20, 27).

We found that the primary cause of lumbar puncture failure in scoliosis patients was the transverse rotation of the lumbar vertebrae. Moreover, the greater the vertebral rotation angle, the higher the likelihood of traditional lumbar puncture failure (20). The lumbar vertebral rotation angle could be measured using CT axial images. Even in cases of severe scoliosis, the spinous process could still be palpable on the body surface. The lumbar vertebral rotation angle could be measured using CT axial images.

Based on the above assumptions, we developed a lumbar puncture method guided by CT scans and precise calculations. The key to this new method is to calculate the puncture angle.

Several tips can aid in performing lumbar puncture using this new approach. Firstly, the actual insertion depth is less than the measured depth, as the needle only needs to pierce the dural sac, not reach its center. Secondly, to facilitate the measurement, the patient's back should be as perpendicular to the bed surface as possible when lying on their side (Figure 1F). The solid blue line in Figure 1F corresponds to the same line shown in Figures 1D, E. The angle between the blue dashed line (perpendicular to the auxiliary line in Figure 1F) and the red puncture path line is the α angle. Thirdly, there are many factors should be considered when selecting the optimal puncture path. In general, puncture is easier with a smaller rotation angle, a shorter insertion depth and a larger dural sac area (14). The L3–L4 intervertebral space is typically preferred. Fourth, patients are advised to lie on the side opposite to the vertebral rotation during puncture, as this position facilitates cerebrospinal fluid drainage by keeping the needle tip higher than the tail. Consistent with previous reports (14), we also find that cobb's angle is not associated with lumbar puncture difficulty. For example, patients 3 and 5 have severe scoliosis, but the spinous process is still palpable, and puncture proceeds smoothly with precise α angle calculation. Although patient 3 also has thoracic segment curvature, it doesn't affect the lumbar puncture.

SMA patients require regular spinal assessment every 6 months or 1 year based on the degree of scoliosis (28). Early pharmacological treatment can slow down the progression of scoliosis, particularly in patients with type II SMA. The progression of scoliosis is relatively slow in type III SMA patients. For skeletally immature patients with a spinal curvature exceeding 50 degrees or those with deteriorating function, surgical intervention is recommended (29). Therefore, in clinical practice, we perform CT scans every 3 years. In fact, these patients have only undergone one CT scan so far.

The small sample size is the primary limitation of this observational study. More complex scoliosis types, such as excessive rotation angles, are encountered in clinical practice. Obesity is another significant factor influencing the success of intrathecal injections. The BMI of patients with severe scoliosis is difficult to measure, and these patients may be unable to maintain the required position.

## Conclusion

Based on 58 lumbar punctures performed on the above 6 patients, we preliminarily confirm the effectiveness and safety of the new intrathecal injection method. This new method requires only one plain CT scan and precise calculations to facilitate bedside, sedation-free intrathecal injection. This technique can be applied and validated in a larger patient population.

## Data Availability

The original contributions presented in the study are included in the article/supplementary material, further inquiries can be directed to the corresponding author.
